# A Social-Emotional Learning Program for Suicide Prevention through Animal-Assisted Intervention

**DOI:** 10.3390/ani11123375

**Published:** 2021-11-25

**Authors:** Alexander Muela, Nekane Balluerka, Eneko Sansinenea, Juan Manuel Machimbarrena, Jon García-Ormaza, Nekane Ibarretxe, Ane Eguren, Patxi Baigorri

**Affiliations:** 1 Department of Clinical and Health Psychology and Research Methodology, University of the Basque Country UPV/EHU, 20018 San Sebastián, Gipuzkoa, Spain; nekane.balluerka@ehu.eus (N.B.); eneko.sansinenea@ehu.eus (E.S.); juanmanuel.machimbarrena@ehu.eus (J.M.M.); egurenane@gmail.com (A.E.); 2 Biocruces Bizkaia Health Research Institute, 48903 Bilbao, Bizkaia, Spain; jon.garciaormaza@osakidetza.eus; 3 Bizkaia Mental Health Network, Osakidetza Basque Health Service, Zamudio Hospital, 48170 Zamudio, Bizkaia, Spain; 4 Department of Neurosciences, University of the Basque Country UPV/EHU, 48940 Leioa, Bizkaia, Spain; 5 Agintzari Cooperative Society of Social Initiative, 48014 Bilbao, Bizkaia, Spain; lokarriiaa@outlook.com; 6 Department of Basic Psychological Processes and Development, University of the Basque Country UPV/EHU, 20018 San Sebastián, Gipuzkoa, Spain; patxi.baigorri@ehu.eus

**Keywords:** young suicide, suicide prevention, SEL programs, animal assisted interventions, animal assisted therapy, human–animal bond

## Abstract

**Simple Summary:**

Youth suicide is a global public health problem. According to data from the World Health Organization (WHO), suicide is the fourth leading cause of death in the age group between 15 and 29 years, after injuries due to traffic accidents, tuberculosis, and interpersonal violence. For this reason, the reduction of mortality by suicide is one of the WHO’s priority objectives. Here we describe a pilot study evaluating the OverCome-AAI program, a pioneering animal-assisted intervention for preventing suicidal behavior. After the intervention, the young people showed reductions in suicidal behavior and non-suicidal self-harm, as well as a greater predisposition to seek help. Mental pain was less intense, although no changes in symptoms of hopelessness or depression were found. The results of this pilot study suggest that the inclusion of specially prepared and trained animals can promote socio-emotional learning for preventing suicidal behavior in high-risk populations.

**Abstract:**

The aim of the study was to carry out a pilot implementation and evaluation of the OverCome-AAI program, a pioneering program for the prevention of suicidal behavior through animal-assisted interventions for young people with high risk factors for suicidal behavior. The study sample consisted of 30 adolescents (11 boys and 19 girls) aged between 14 and 17 years (Mean age = 15.50, *SD* = 1.60) from the Basque Country (Northern Spain). After the intervention, subjects presented reductions in suicidal ideation, suicide plans, and non-suicidal self-harm, as well as a greater predisposition to seek help. A reduction in the intensity of mental pain was also found, although no differences were observed in indicators of hopelessness and depression. The preliminary results obtained in this pilot study suggest that the OverCome-AAI program may be effective in reducing suicidal behavior and non-suicidal self-harm in young people in residential care who present high risk factors for suicide.

## 1. Introduction

Suicide is a public health problem that affects all countries, ages and genders. According to epidemiological data, the global age-standardized suicide death rate is nine people per 100,000 inhabitants, although the rate varies widely across countries: from two suicides per 100,000 people to more than 80 [1]. In terms of geographical distribution, suicide deaths exceed the global average in Africa (11.2 per 100,000), Europe (10.5 per 100,000) and South-East Asia (10.2 per 100,000). The lowest rates are found in the Eastern Mediterranean Region, with 6.4 cases per 100,000 inhabitants. Men have a suicide rate of 12.6 per 100,000 people, while women have a rate of 5.4; thus, the rate is 2.3 times higher in men than in women, and the proportion is higher in high-income countries [1].

According to data from the WHO [1], suicide is the fourth leading cause of death in the age group between 15 and 29 years, after injuries due to traffic accidents, tuberculosis, and interpersonal violence. As far as the age of onset and course are concerned, suicide is rare in childhood and puberty. In the 10–14 year age group, most suicides occur between the ages of 12 and 14. After puberty, suicide rates increase with age and then stabilize in early adulthood.

Several recent meta-analyses and systematic reviews [2,3] estimate the suicide rate for both sexes and for young people aged 15 to 19 at between 3.8 and 3.9 per 100,000 people, although as occurs in other age ranges, considerable variations have been found between the countries analyzed. The WHO [1] warns that the official global figures are probably lower than the true rates, since only 80 of the 183 WHO Member States for which estimates were published in 2021 had civil registries with good quality data; most of the countries without quality data were low- and middle-income countries [1]. Countries such as India, China, and most African nations have not yet developed reliable recording systems [2]. The WHO estimates that the suicide rate in countries that do not have adequate registration systems represents approximately 71% of the global annual total [4]. This underreporting is heightened by the fact that many probable deaths by suicide are not classified as such (either out of fear of social stigma, or because forensics categorize the cause of death inaccurately); in fact, estimates of underreporting range between 10% and 50%, due to the lack of a specific definition of suicide and the absence of a standardized classification system [5]. It is assumed that this phenomenon of misclassification is more marked in countries where suicide is an illegal practice or violates religious norms [4].

As is the case with the general data on suicide for all age groups, in adolescence females present a higher rate of suicide attempts (between three and nine times more than males) but males have a higher rate of death by suicide (between three and four times more) [2]. Similarly, girls are more likely to have devised a suicide plan (13.4%) than boys (9.2%) [6].

Suicide in the youth population is a complex and multi-causal phenomenon [6]. It generally occurs when certain stressful life events and mental health factors converge, provoking in the person an experience of hopelessness, social isolation, and despair. Experts confirm that the most important predictor of suicide committed in youth is a history of previous suicide attempts [7]. Likewise, engaging in non-suicidal self-injurious behaviors (e.g., self-cutting, burning, hitting) is considered to be one of the main predictors of making suicide attempts [8]. Suicidal thoughts and non-suicidal self-injurious behaviors have been associated with suicide attempts, however, the majority of adolescents who think about suicide or who self-harm without intending to end their lives will not attempt suicide. Nevertheless, the combination of these two factors suggests that the young person will present a high risk of suicidal behavior [9]. In fact, a recent study [9] found that approximately one in five adolescents (21%) who reported both suicidal thoughts and non-suicidal self-harm made a suicide attempt in the following months, compared with only 1% of those who did not report either of these behaviors. Other variables considered risk factors for suicide include mental health disorders, mental pain, hopelessness and lack of connectivity [9].

Reducing mortality from suicide is one of the priority goals of the WHO [1]. Proof of this is the inclusion of suicide prevention as an indicator in the United Nations Sustainable Development Goals, in target 3.4, in the 13th WHO General Work Program 2019–2023, and in the WHO Mental Health Action Plan 2013–2030. In order to reinforce effective actions for mitigating the impact of suicide, the WHO [10] proposes the establishment of four priority lines of action: limiting access to the means of suicide, interacting with the media for responsible reporting of suicide cases, implementing programs to promote socio-emotional life skills in young people, and promptly identifying, assessing, managing and monitoring people with suicidal thoughts and behaviors.

Several systematic reviews and meta-analyses [11,12] have found that youth suicide prevention programs are effective in reducing suicide risk factors. Early identification of suicidal thoughts and behaviors, and effective care for those at risk, are crucial to ensuring that people receive the care they need and deserve. Prompt action is critical to any strategy for suicide prevention. Therefore, there is a need to develop and implement effective health policies and strategies for the prevention of suicidal behavior in adolescents and young adults and for the early identification and reduction of the most prevalent risk factors.

It is common to classify prevention strategies under three different headings: universal, selective, and indicated. Universal prevention programs for children and adolescents are carried out in the school environment with the aim of raising awareness about suicide. One of the universal programs that has received the most empirical support is the Signs of Suicide (SOS) program in the US, aimed at adolescents between 13 and 18 years of age and taught over a couple of days by the students’ usual teachers. Several studies (e.g., [13]) have shown that this program reduces notifications of suicide attempts by 40% and that it increases participants’ readiness to intervene in situations in which a friend may be exhibiting signs of suicidal behavior, and to seek help if they themselves present indicators that are associated with suicide or depressive symptoms.

Selective prevention is a type of early intervention that is aimed at children and adolescents presenting risk factors for suicide. It is generally implemented in schools and community health and psychosocial care services. The objective of selective prevention programs is to train members of these communities to identify young people who are at risk of suicide and to refer them to specialized health services [7]. A successful example is Question, Persuade, and Refer (QPR), a gatekeeper program designed to train teachers and other members of the school community to identify adolescents at risk of suicide. The aim of the QPR is to educate facilitators about the myths surrounding suicide and suicidal behavior, the warning signs that they must be able to identify and the resources that they can propose to the person affected. The program also trains community facilitators in the skills needed to ask young people about their suicidal thoughts and/or intentions, persuade them to get help, and accompany them to specialized health services. The QPR is included in the National Registry of Evidence-based Programs and Practices of the Substance Abuse and Mental Health Services Administration and among the programs with evidence of effectiveness of the Suicide Prevention Resource Center. The QPR has been shown to increase gatekeepers’ knowledge of suicide, their ability to detect young people at risk, and their readiness to intervene [14].

Another program that has obtained good results in preventing suicide in the child and adolescent population is the ASIST (Applied Suicide Intervention Skills Training) program, a 14 h training workshop held over two consecutive days which aims to prevent the immediate risk of suicide. The study by Gould et al. [15] with the National Suicide Prevention Lifeline in the US found that people attended by professionals who had been trained on the ASIST program showed fewer feelings of depression, felt less overwhelmed, and were more hopeful of overcoming the situation they were experiencing. A positive effect has also been found with adolescents in school environments. In fact, it has been observed that school staff (teachers, counselors and administrative staff) who receive training on the ASIST program are more able to detect cases of suicide and show better competence and greater confidence to respond appropriately to people at risk of taking their lives [16].

Indicated prevention is aimed at reducing the risk of suicidal behavior in minors with high risk factors such as depression, psychiatric comorbidity, suicidal thoughts, and suicidal planning or in those who have already displayed serious suicide behaviors such as suicide attempts or non-suicidal self-harm [7]. Indicated prevention requires multidisciplinary services that offer assistance, care and follow-up programs.

The present study focuses on the prevention of suicidal behavior in the “indicated” population, specifically in adolescents and young people who are in residential child care. Many of these young people present high risk factors for suicidal behavior such as high indicators of psychopathology, traumatic life history or low psychosocial adjustment. Previous research has shown a higher prevalence of suicidal ideation in young people in residential child care away from their biological family than in their peers in non-care populations (24.7% versus 11.4%) [17]. In addition, young people in foster care have higher rates of suicide attempts (between three and nine times higher) than those never placed in foster care [18]. Few studies to date have assessed rates of completed suicide: those carried out have found that, among young people under protection measures (including those in residential care) the death rate from suicide is between two and six times higher than in the general population [19,20].

The present article describes the pilot implementation and evaluation of the OverCome-AAI program, a pioneering program for the prevention of suicidal behavior through animal-assisted interventions (AAI). AAIs are structured, goal-oriented activities that intentionally incorporate animals for the purpose of obtaining therapeutic benefits and improvements in health and well-being. These interventions include animal-assisted education, animal-assisted activities, and animal-assisted therapy.

AAI is an emerging area in the field of psychosocial intervention. Several meta-analyses [21,22,23] and review studies [24,25] have demonstrated the positive effects of AAIs in the educational and healthcare contexts. AAIs have been used in the treatment of children and adolescents with learning disabilities, autism, people with serious illnesses (HIV virus, multiple sclerosis, cancer, and in palliative care), adolescents and adults with psychiatric problems, people with disabilities, people with aphasia or other language problems, elderly people with depressive symptoms or with Alzheimer’s disease, and so on.

Human beings are believed to have an innate need to understand nature and to relate to it. This relationship has an influence on their cognitive level, health, and well-being, a concept known as biophilia [26]. Several authors [27,28] hold that one of the keys to the success of AAI is the creation of the human–animal bond. Archer and Ireland [29] and Kurdek [30] found that owners build up a close affective attachment with their pets; animals provide them with a secure base and a source of emotional support and well-being, as well as a safe haven and source of relief in difficult times [31].

With regard to this human–animal bond, there is evidence that interacting with animals reduces symptoms of anxiety and depression [32], alleviates pain [33] and improves physiological markers of well-being and response to stress [21]. Interaction with animals has been associated with a reduction in systolic blood pressure and an increase in diastolic blood pressure [21], a reduction in blood pressure [34], an increase in oxytocin secretion [35] and reductions in levels of cortisol [36], epinephrine and norepinephrine, and cardiopulmonary pressure [37]. Human–animal interactions have also proved useful in the hospital setting where they have been associated with reduced stress levels in hospitalized patients and in those undergoing surgery [38].

Several recent studies with children and adolescents who have suffered traumatic experiences have suggested that the use of AAIs improves health and prevents risky behaviors [39]. Animals behave spontaneously; they are always available for interaction, they do not prejudge, they provide unconditional love, and they are loyal and affectionate. In general, they are not intimating, and their multisensory characteristics make them uniquely suited to interventions with children and adolescents who have suffered traumatic experiences. These innate elements favor a climate of security and trust and help to establish and consolidate the therapeutic relationship. They encourage spontaneous communication, motivate patients to engage with the therapeutic process and reduce the feelings of rejection and stigmatization, which are all key elements of a successful intervention process [40]. In general, AAIs have been shown to reduce symptoms associated with post-traumatic stress, depression and anxiety [41,42]. When carried out with young people in the child protection system, AAIs promote psychosocial adaptation and resilient behavior [4,41], and also enhance attachment security [27].

Due to the innate characteristics of animals, AAIs facilitate the therapeutic process in children and adolescents who have suffered childhood trauma and maximize its benefits [41,42]. AAIs also increase motivation and adherence to treatment [43]; these are key issues in the management of people who have suffered traumatic events, given the very high dropout rate [22].

Taking into account what has been indicated so far, we expected that after the intervention the adolescents would present reductions in suicidal behavior and non-suicidal self-harm and improvements in indicators that are closely related to suicidal behavior such as mental pain, hopelessness, and depressive symptoms.

## 2. Materials and Methods

### 2.1. Participants

The study sample consisted of 30 adolescents (11 boys and 19 girls) aged between 14 and 17 years (mean age = 15.50, *SD* = 1.60) from the Basque Autonomous Community in Spain. All participants were in residential child care, under the protection of the competent authorities. Most presented either suicidal behavior or risk factors for suicidal behavior: in fact, 53.3% had previously made suicide attempts, 70% had suicidal ideation in the six months prior to starting treatment, and 60% had devised a plan to end their life. Furthermore, 80% had engaged in non-suicidal self-harm in the last six months and 76.7% had a psychiatric diagnosis of a mental health disorder and were receiving psychopharmacological treatment. Girls showed higher rates of previous suicide attempts (61.1% versus 36.4%), suicidal ideation (83.3% versus 45.5%), suicide plans (77.8% versus 36.4%) and non-suicidal self-harm (89.9% versus 63.6%). Three participants (one girl and two boys) did not complete the intervention, two due to hospital admission and one who escaped from the residential center.

### 2.2. Instruments

The scale for the evaluation of suicidal behavior in adolescents (SENTIA, the original Spanish version) [44] is a self-report that consists of 16 items with a yes/no response format. It assesses the presence of suicidal behavior through three dimensions or subscales: suicidal ideation (e.g., “Have you ever had ideas about taking your life?”), suicidal communication (e.g., “Have you told anyone that you want to take your own life?”), and planning (e.g., “Have you thought of a plan to end your life?”). It also measures whether the adolescent engages in non-suicidal self-harm (e.g., “Have you ever hurt yourself—self-harming, cutting yourself, etc.—without meaning to kill yourself?”) and whether he/she seeks help for his/her suicidal ideation (e.g., “Have you done anything to make others understand that you wanted to take your life?”). SENTIA has been validated in young Spanish populations; it has shown good psychometric properties in terms of its internal structure validity, and it has also shown evidence of validity based on positive relations between its dimensions and symptoms of depression and other emotional and behavioral problems [44]. The internal consistency in the present sample was 0.93 (Cronbach’s alpha).

The Spanish version of the eight-item Patient Health Questionnaire (PHQ-8) [45,46]. The PHQ-8 consists of eight items (e.g., “Feeling down, depressed, or hopeless”) that assess the presence of depressed mood, anhedonia, sleep problems, fatigue, changes in appetite or weight, feelings of guilt or worthlessness, difficulty concentrating and feelings of laziness or worry (e.g., “Feeling down, depressed, or hopeless”). The items refer to the past 14 days and are answered on a four-point Likert scale, from 0 (never) to 3 (almost every day): 0 (never), 1 (several days), 2 (more than half the days), and 3 (almost every day). Thus, total scores range from 0 to 24. The authors [46] suggest five levels of severity: minimal (total score, 0–4); mild (total score, 5–9); moderate (total score, 10–14); moderately severe (total score, 15–19); and severe (total score, 20–24). A score of 10 or above is frequently used as a cut-off point to identify patients with major depression. This assessment tool has been validated in a Spanish-speaking population, showing good indicators of construct validity, discriminant validity, and factor validity [45]. Internal consistency in the present sample was 0.79 (Cronbach’s alpha).

The Spanish version of the Psychache Scale (PS-E) [47,48]. The PS-E assesses mental pain. It includes 13 items valued on a Likert scale of 5 response options (e.g., “I can’t take my pain any more”). From item 1 to item 9 the response options range from 1 = “never” to 5 = “always”,” (e.g., “I feel psychological pain.”), and from item 10 to item 13 they range from “strongly disagree” to “strongly agree”.” (e.g., “I can’t take my pain any more”). The higher the score, the more intense and frequent (i.e., less bearable) the perception of mental pain. This scale has been validated in a Spanish population showing good internal structure validity and evidence of validity based on positive correlations with other related variables such as depression, hopelessness, suicidal ideation and suicidal risk [47]. In the present sample, the Cronbach’s alpha coefficient of internal consistency was 0.95.

The Spanish version of the Beck Hopelessness Scale-Short Form (BHS-SF) [49]. The BHS-SF measures the sensation of hopelessness during the last week (e.g., “My future looks dark to me”). Items are rated as true or false and total scores can range from 0 to 4. The BHS-SF is a four-item version of the widely used BHS [50], which has been validated with a Spanish population [51], and it shows good psychometric properties [50]. Internal consistency in the present sample was 0.80 (Cronbach’s alpha).

The questionnaire on the learning acquired in the intervention and its perceived usefulness was created ad hoc for this study. It consists of seven open questions that record information on participants’ expectations (e.g., “Think about the reason why you agreed to take part in the program. What were your expectations? What has motivated you to continue?”) and on the aspects they see as the most useful for avoiding suicidal behavior both during treatment (e.g., “Of all the things you have learned, what is the most useful to you? Has it helped you to avoid engaging in suicidal behavior during this time?”) and in the future (e.g., “Of all the things you have learned, what do you think will help you the most in avoiding suicidal behavior in the future?”). It also asks about the obstacles to applying the knowledge acquired during treatment and also in the future (e.g., “What do you think will be the biggest obstacles you will encounter with regard to applying what you have learned?”). Finally, they are asked to rate how far the intervention program has improved their ability to cope with suicidal crisis situations, on a scale of 1 to 10 points.

### 2.3. Procedure

First, consent to carry out the study was obtained both from the Child Protection Services and from the adolescents, and a set of sociodemographic data was collected. After confirming the inclusion criteria (age between 13 and 17 years old, being in residential child care, presenting risk factors for suicidal behavior) and the exclusion criteria (serious conduct disorder with aggression towards people and animals, psychotic disorders, substance dependence and aversion to animals), the evaluation questionnaires were administered (pretest). The AAI-based program for suicide prevention was then applied, and finally, two weeks after completion of treatment, the assessment instruments were re-administered (post-test). The questionnaire on learning and satisfaction with the intervention was administered after the last session, at the end of the treatment. This process was replicated under the same conditions six times with the different groups during the 2021 calendar year. Between four and six participants were included in each intervention group. As the implementation of the program coincided with the COVID-19 pandemic, all the health security measures stipulated by the Spanish Ministry of Health were applied, such as hand washing and the use of masks. It is important to say that the research team undertook to attend to all those adolescents that presented risk factors for suicidal behavior and therefore it was not possible to have a control group.

The OverCome-AAI intervention program is group-based and consists of six 90-min sessions held weekly. It focuses on various interpersonal and emotional regulation skills for preventing suicidal behavior. Each week, participants receive a recording in which they daily practice the emotional regulation technique presented during the face-to-face session with the help of a member of the residential care center staff. Table 1 outlines the treatment program, the competencies to be achieved, and the emotional regulation techniques that are practiced.

The aim of the first session is to help the young person understand what suicidal behavior consists of. Reliable information on suicide and non-suicidal self-harm is offered, in an attempt to dispel the multiple social myths, misconceptions and misinformation that surround the issue. In the second session, the risk factors associated with suicidal behavior in adolescence and youth are studied—for example, being the victim of bullying, the consumption of alcohol and drugs, or mental health problems—and the importance of promoting health is emphasized. In the third session, the skills needed to detect the warning signs of suicidal crisis are studied, both psychological signs (mental pain, the perception of being a burden, hostility towards oneself, depression, etc.) and physical and behavioral signs (agitation or irritability, muscle tension, fatigue, sleep disturbances, etc.). In the fourth session, special attention is paid to the warning signs of lack of connectivity and negative self-criticism, and prevention strategies and coping skills are discussed and applied. In the fifth session, participants draw up a safety plan: that is, a personalized list of coping strategies and sources of support that they can use or turn to before or during a suicidal crisis. This personalized safety plan consists of procedures to help the young person to recognize the warning signs associated with an impending crisis, the internal coping strategies that can be deployed in this situation, and the social distractors (both places and people) who can help reduce suicidal thoughts. The plan also includes the contacts of close people and health professionals or organizations who can offer assistance in crisis situations. In the last session, the intervention program is assessed, community resources for suicide prevention and crisis care are described, and the safety plan is redefined and consolidated as an instrument for preventing suicide attempts.

The professional team in charge of the intervention comprised two clinical psychologists supported by a veterinarian trained in animal-assisted interventions. Two Labrador Retrievers, six and two years old, were used for the intervention. They were selected for their genetic inheritance (docility, sociability and great flexibility in the face of stressful stimuli) and because, thanks to their training, they were calm, had a genuine interest in interaction with humans, and were adaptable to new situations. The dogs were selected by the veterinarian team. Both were trained prior to the intervention and the necessary measures were followed at all times to safeguard their welfare. The animals were vaccinated and underwent external and internal deworming prior to the interventions, in order to guarantee the safety of the participants.

The program sought to capitalize on the human–animal bond as an ecological adjustment and therapeutic instrument [52], and was carried out at all times under the guidance of the vet. The dogs’ role was mainly to encourage socialization and provide re-assurance; their presence facilitated communication and fostered the relationships between participants and between participants and the therapist. For example, when a participant shared difficult-to-express emotions associated with a suicide crisis situation, the animal acted as a multi-sensory mediator, providing both a sense of calm and physical contact, thus aiding emotional regulation. The dogs also served as distractors in emotional crisis situations. For example, one of the objectives of the program’s safety plan devised in the fifth session is that the young people should learn to apply coping techniques or strategies that they could use on their own to control negative thoughts and feelings and the decompensation that often follows on from an escalation of the behaviors that trigger a suicide attempt. For this purpose, the young people were asked to imagine some of the negative thoughts that might trigger a suicide crisis (e.g., “nothing can help me solve my problems”). Next, they practiced distracting recreational exercises involving the animal (feeding the dog, giving commands to sit or to offer a paw, taking the dog for walks, guiding the dog to pass beneath their legs as a sign of trust, etc.). These exercises showed participants how effective distracting techniques can be when they identified the warning signs of a suicide crisis. Based on these experiences, participants were asked to select and work on personal distraction strategies for regulating their emotions.

In addition, the animals were used to arouse feelings of tenderness and compassion, which helped to work on the techniques associated with compassion-focused therapy: that is, promoting the ability to generate supportive, kind, validating and under-standing emotions. This is very important in young people who present suicidal behavior, since they tend to feel very hostile towards themselves and present high levels of self-criticism, shame and self-contempt [53].

Finally, the dogs also provided emotional support. They were trained to offer positive affection, especially with people presenting anxious emotional states. This allows the participants to engage in a genuine caring relationship and to develop a greater sense of trust in the therapeutic process, which, in turn, is likely to prompt them to seek assistance in situations of suicide crisis. This is a crucial step because, as noted above, young people with suicidal ideation and behavior lack faith in healthcare professionals and are generally very reluctant to seek help.

### 2.4. Data Analysis

The nonparametric McNemar test was used to establish whether there were changes in suicidal behavior, non-suicidal self-harm, and seeking help in the intervention group after participating in the OverCome-AAI suicide prevention program. To examine whether the OverCome-AAI program influenced indicators of depression, mental pain and hopelessness, the group’s mean pre-test and post-test scores were compared. After verifying that the assumptions for the application of parametric tests were fulfilled, the Student’s t test was used to examine whether there were statistically significant differences between the means in the different criterion variables. The effect size associated with the mean differences was calculated using Cohen’s d.

All data analyses were carried out using SPSS software (IBM Corp. Released 2019. IBM SPSS Statistics for Windows, Version 26.0. Armonk, NY, USA).

## 3. Results

First, it should be stressed that none of the participants made a suicide attempt over the course of the OverCome-AAI program. Regarding the changes in suicidal behavior during the program, statistically significant differences were observed in suicidal ideation (χ^2^_McNemar_ = 6.75; *p* < 0.05) and in plans to kill oneself (χ^2^_McNemar_ = 12.07; *p* < 0.05). Changes were also found in non-suicidal self-harm (χ^2^_McNemar_ = 12.07; *p* < 0.05). With a confidence level of 95%, the proportion of adolescents presenting suicidal ideation decreased after the intervention by between 0.12 and 0.62 points. The proportion of those engaging in non-suicidal self-harm presented similar falls (between 0.25 and 0.79 points). Likewise, statistically significant differences were found (χ^2^_McNemar_ = 8.10; *p* < 0.05) in the perception of being able to ask anyone for help when having suicidal thoughts, with notable increases of between 0.14 and 0.60 points after the intervention. Regarding requests for help, no statistically significant differences were found after the intervention. However, in the post-test evaluation 85% of the young people who had presented suicidal ideation had requested help due to their suicidal thoughts, compared with only 38.1% prior to the intervention. Finally, no significant differences were found between boys and girls in the post-test in suicidal behavior, although girls continued to present higher rates of suicidal ideation (35.3% versus 11.1%) and of non-suicidal self-harm (29.4% versus 22.2%).

Table 2 shows the mean scores and standard deviations of the treatment group in the pretest and posttest evaluations for the indicators of depression, mental pain and hopelessness.

After the intervention, significant differences were observed in the treatment group in mental pain (t (26) = 2.640; *p* = 0.014; d = 0.51), but no differences were found in either depression or hopelessness. In both these cases the effect sizes associated with the differences between the means were small (Cohen’s d = 0.29 and 0.12 respectively).

Regarding the questionnaire on the learning acquired in the intervention and its perceived usefulness, most of the young people reported that they had been obliged to participate and that their initial expectations were low. However, they also stated that the program had helped them to cope better with the suicidal crises, that the sessions had been more dynamic than they had expected, and that the interaction with the dogs had represented a strong motivation to continue. As for the emotional regulation exercises, participants said that they had found it difficult to practice them every day and that, although the exercises helped alleviate their emotional stress, they were unlikely to apply them autonomously on a regular basis in the future. In this respect, they considered the help and follow-up received from the residential care center staff to be important. With regard to the socio-emotional strategies for preventing suicide, after the intervention many participants reported an improvement in their ability to detect psychological and physical-behavioral warning signs. The tools that had helped them the most to avoid suicidal behavior during treatment were the emotional regulation techniques and the safety plan, which they learned to execute step by step. As far as preventing suicidal behavior in the future was concerned, they stated that the most important step forward was the change in their attitude towards seeking help. Finally, their evaluations of the program showed that it had improved their ability to cope with suicidal crisis situations (mean rating 7.89, *SD* = 1.34).

As regards attendance, 91% of the participants who completed the treatment attended all the sessions and regularly practiced emotional regulation exercises outside the sessions. None of the other participants missed more than one session, and they also practiced emotional regulation techniques frequently.

## 4. Discussion

The main aim of this pilot study was to implement and evaluate the OverCome-AAI program, a pioneering program for the prevention of suicidal behavior in young people that incorporates animal-assisted interventions.

Assessing the effect of the program on suicidal behavior, after the intervention, participants presented reductions in suicidal ideation, suicide plans and non-suicidal self-harm. These data agree with the results of other socio-emotional learning suicide prevention programs aimed at young populations, although it should be noted that most of the other programs have been carried out in the school setting [54,55,56]. This study is novel in that the prevention program was tested in adolescents in a residential child care environment with high risk factors for suicide, and that the program incorporated animal-assisted interventions.

A positive effect was found in a dynamic variable associated with suicide attempts, namely, the intensity of mental pain. Mental pain is a highly disturbing psychological state characterized by an internal experience of negative emotions such as shame, anguish, guilt, humiliation, loneliness and fear [57]. Therefore, reducing the intensity of mental pain is vital to ensure that the young person does not move on from suicide ideation to a suicide attempt, and this positive effect is greatly reinforced if his/her feelings of hopelessness are also attenuated. In the present study, however, no changes were obtained in hopelessness.

Similarly, it is important to note that no changes were observed in depressive symptoms. This result supports the idea that socio-emotional learning programs do not tend to modify indicators of psychopathology in young people, or that at best their influence is small: this has also been observed in universal prevention social-emotional learning programs carried out in school environments [58,59], although some specific suicide prevention programs have obtained positive results in reducing depressive symptoms [55]. With regard to the present program, this result may indicate that suicidal ideation and non-suicidal self-harm are modified even though indicators of depression do not present major changes. However, this statement must be treated with extreme caution since indicators of depression were only measured with a screening method.

A notable benefit of the intervention program was that participants felt more trusting of their immediate environment and were more positive with regard to seeking help for their suicidal behavior. This perception that they could ask for help if they needed it may reflect an increase in their confidence to turn to others at times of high emotional suffering, manifested in suicidal ideations and/or non-suicidal self-harm. We consider this to be a very important contribution of the OverCome-AAI program, thanks to its focus on developing the ability to detect suicide warning signs and to apply a suicidal behavior safety plan. We consider that our adolescents have probably internalized the importance of asking for help, and the stigma they may have felt with regard to engaging in suicidal behavior may have receded. Indeed, our results corroborate those of other programs such as the Sources of Strength program [60] and Signs of Suicide [12].

Taking the results together, the OverCome-AAI suicide prevention program can be a promising intervention program to positively influencing on the three main domains of suicide prevention in young people: knowledge, attitudes and behavior [61]. First, it broadens their knowledge about the myths surrounding suicide, the scientific data about suicide, the risk factors and the alarm signals for detecting situations of suicide risk. As for attitudes, the program achieved positive results especially with regard to help-seeking and social perception of stigma. Finally, as far as behavior is concerned, it helps to improve young people’s emotional regulation strategies. However, further studies are needed to verify these effects, especially with regard to knowledge and attitudes.

The present study has some limitations. The first is the small sample size and the fact that it is not representative with regard to sex, since the sample comprised more females than males. Being a pilot study, the conclusions should be interpreted with caution since its statistical power is low, and the results obtained cannot be generalized to other settings. Therefore, this study must be replicated with a sufficiently representative sample to allow population inferences to be made. Likewise, in order to isolate the effect of the OverCome-AAI program from other variables, future studies should add a control group that does not take part in the program.

It would also be useful to apply other multi-informant measures of the variables under study. For example, it would be interesting to introduce the impressions of school or residential care staff in the study. Furthermore, the adolescents who undergo the treatment should be followed up over longer periods of time in order to establish whether the positive effects are maintained. This follow-up should also study the application of both the emotional regulation exercises and the safety plan over time, and the possible relationships with the frequency of suicidal crises and non-suicidal self-harm.

Finally, as recent research into suicide has advocated [62], future studies of this intervention program should apply qualitative methods to determine its effect. It would be interesting to use ethnographic methods such as participant observation to study attitudes to suicidal behavior and the social interactions of the young people undergoing the treatment. An interpretative phenomenological analysis might also be able to determine whether the intervention program has had a preventive effect on the social learning of suicidal behavior when a young person at a residential center presents non-suicidal self-harm or makes a suicide attempt. Finally, narrative and biographical methods could also be used to better understand how young people who have received treatment manage stressful life situations, and how they cope with the warning signs associated with a suicide crisis.

## 5. Conclusions

The preliminary results obtained in this pilot study suggest that the OverCome-AAI program may be effective in reducing suicidal behavior and non-suicidal self-harm in young people in residential care who present high risk factors for suicide. The inclusion of animals in the OverCome-AAI program represents an added value and offers a promising alternative to the more traditional social-emotional learning programs. One of the main consequences of the presence of animals is the high adherence to treatment and the low dropout rate, something that standard psychotherapeutic procedures find hard to achieve [41]. Including specially trained animals to facilitate social-emotional learning interventions maximizes their impact, as previous studies have shown [41,42].

## Figures and Tables

**Table 1:** Summary of the OverCome-AAI program.

Session	Competencies	Emotional Regulation Skill
Facts, beliefs and myths about suicide.	Identify reliable facts about suicide. Identify myths and realities about suicide. Know what defines suicide and suicidal behavior. Learn not to treat suicide as a taboo subject: talking about suicide prevents it.	Progressive muscle relaxation
2. Risk factors and protective factors of suicide.	Know the risk factors and protective factors for suicide. Acquire skills to recognize suicide risk situations.	2. Respiratory energization
3. Warning signs of suicide	Identify suicide warning signs and assess the importance of responding to these warning signs and intervening. Acquire skills for the management of mental pain. Promote personal self-care.	3. Diaphragmatic breathing
4. Connectivity, self-pity and negative criticism.	Develop protective suicide prevention skills through the development of connectivity and a sense of life. Develop skills to handle hostility towards oneself.	4. Experiential acceptance (Mindfulness)
5. Suicide risk safety plan	Establish a safety plan in the case of suicide alarm symptoms.	5. Prudent distance (Mindfulness)
6. Closure and additional help resources	Identify additional resources in situations of suicide risk. Learn to seek and request help in situations of suicide risk.	6. Focusing on difficult emotions

**Table 2:** Mean scores and standard deviations in the treatment group in indicators of depression, mental pain and hopelessness before and after the intervention.

	Evaluation	Mean	SD	N
Depression	Pretest	11.10	5.66	30
	Post-test	13.55	6.75	27
Mental pain	Pretest	36.13	14.86	30
	Post-test	30.30	13.20	27
Hopelessness	Pretest	0.83	1.26	30
	Post-test	0.78	1.28	27

## Data Availability

The data presented in this study are available on request from the corresponding author.
